# Pulseless Electrical Activity: Echocardiographic Explanation of a Perplexing Phenomenon

**DOI:** 10.3389/fcvm.2021.747857

**Published:** 2021-11-05

**Authors:** David C. Parish, Hemant Goyal, Erskine James, Francis C. Dane

**Affiliations:** ^1^Department of Medicine, Mercer University School of Medicine, Macon, GA, United States; ^2^The Wright Center for Graduate Medical Education, Scranton, PA, United States; ^3^Mercer University School of Medicine, Macon, GA, United States; ^4^Department of Internal Medicine, Atrium Health Navicent, Macon, GA, United States; ^5^Department of Psychology, Radford University, Radford, VA, United States

**Keywords:** PEA, pulseless electrical activity, Ventricular Fibrillation, cardiac arrest, death & dying

## Abstract

Pulseless electrical activity (PEA) is considered an enigmatic phenomenon in resuscitation research and practice. Finding individuals with no consciousness or pulse but with continued electrocardiographic (EKG) complexes obviously raises the question of how they got there. The development of monitors that can display the underlying rhythm has allowed us to differentiate between VF, asystole, and PEA. Lack of clear understanding of the emergence of PEA has limited the research and development of interventions that might improve the low rates of survival typically associated with PEA. Over 30 years of studying and practicing resuscitation have allowed the authors to see a substantial rise in PEA with variable survival rates, based on the patients' illness spectrum and intensity of monitoring. This paper presents a small case series of individuals with brain death whose family members consented to the echocardiographic observation of the dying process after disconnection from life support. The observation from these cases confirms that PEA is a late phase in the clinical dying process. Echocardiographic images delineate the stages of pseudo-PEA with ineffective contractions, PEA, and then asystole. The process is contiuous with none of the sudden phase shifts seen in dysrhythmic events such as VF, VT or SVT. The implications of these findings are that PEA is a common manifestation of tissue hypoxia and metabolic substrate depletion. Our findings offer prospects for studies of the development of interventions to improve PEA survival.

## Introduction

Pulseless electrical activity (PEA) is a phase in the dying process of humans and animals which is poorly understood by researchers and physicians involved in resuscitation research and practice (1). This is problematic because the percentage of dying patients who are discovered in PEA in in-hospital and pre-hospital studies is now consistently higher than that of Ventricular Fibrillation (VF) (2, 3). PEA has a poor prognosis and no unifying accepted definition. A National Heart, Lung, and Blood Institute Workshop report defined PEA as “a syndrome characterized by an absence of a palpable pulse, in an unconscious patient with an organized electrical activity other than ventricular tachyarrhythmias on EKG,” which is vague but functional (4). The rise in PEA and decline in VF is related to earlier diagnosis and intervention in patients with heart disease, which is a manifestation of change in frequency and severity of disease over time (5, 6). This data calls for a more precise understanding of PEA and increased research regarding potential interventions into reversibility.

Researchers have used cardiac ultrasonography during PEA resuscitation. Studies have identified patients with severe hypotension without clinically detectable pulses but with residual cardiac activity (pseudo-PEA) and ones with absent blood pressure (BP) and no cardiac wall motion (true PEA) (7, 8). Visualization of the heart by 2D-Echocardiogram (2D-ECHO) during resuscitation has been shown to have prognostic value. Patients with evidence of cardiac kinetic activity (pseudo-PEA) have better survival than those with no evidence of contraction (PEA) (8). However, assessment of PEA within human resuscitation efforts is limited by the uncertainty regarding when the pulse was lost, the time required to deploy devices such as 2D-ECHO during resuscitation, and the reality that PEA is present for only seconds to minutes and emerges only late in the dying process (9). In addition, the observation periods have been brief and affected by chest compressions, ventilation, and infusion of vasopressors. For many years, these limitations have prevented determining whether pseudo-PEA and PEA were distinct entities or earlier and later phases of the dying process.

The objective of this prospective study was to describe the patterns through which the heart declines in dying patients through PEA to asystole and assess similarities between animal and human death physiology. For this purpose, we selected a model of clinical death in which no resuscitation would be chosen. In addition, a clearly identified time for the discontinuation of support was available to ensure that the process could be followed continuously.

## Methods

The study was a prospective, observational case series. Following approval from the Institutional Review Boards of Mercer University School of Medicine and The Medical Center/Navicent Health, patients who met clinical criteria for brain death and were not candidates for organ transplants were recruited. Family members were approached, and informed consent was obtained. The protocol consisted of monitoring BP and heart rate to asystole by the placement of a 2D-ECHO probe on the chest before the ventilator was disconnected and following the heart contractions to cessation. No other changes in the care of the patient were implemented; family members were encouraged to be present during the period. An echocardiography technician was present at the patient's bedside at the designated time and identified the best view to evaluate the heart function. Timing of cardiac monitoring, blood pressure detection, and the 2D-ECHO were correlated by researchers who were present throughout the period from ventilator disconnection to asystole.

## Results

Three patients were prospectively included in this study to demonstrate our hypothesis. The patients' demographics and their admission diagnoses are noted in Table 1.

**Table 1:** Patient demographic and admission diagnoses.

	Age range^*^ ** (in years)**	**Sex**	**Race**	**Admission diagnosis**
Patient 1	45–50	Female	Caucasian	Intracerebral hemorrhage (ICH)
Patient 2	70–75	Male	Asian	ICH
Patient 3	60–70	Female	Caucasian	Subarachnoid hemorrhage

* *The patients' age were changed into “range” to avoid the indirectly identifiable data*.

A total of four cardiologists reviewed the echocardiographic recordings, and their observations were summarized by a cardiologist and author (EJ). Patient 1 initially had mild cardiomyopathy with left ventricular ejection function (LVEF) of approximately 45% at the time of withdrawal of life support. There was a gradual decline in left ventricular (LV) systolic function over the next 10 min until the development of PEA, at which time LVEF was ~15%. LV end-diastolic volumes decreased more rapidly, progressing from a left ventricular end-diastolic dimension (LVEDd) of 40 mm at the cessation of life support to an LVEDd of 25 mm at 7 min after cessation, where it remained through the initiation of PEA. Once in PEA, LV systolic function remained poor, and the diastolic filling was a shortened rapid ventricular filling phase and atrial contraction, with a markedly extended diastasis period (Supplementary Video 1).

Patient 2 had normal LV systolic function initially with an LVEF of 65%; LV volumes were normal with an LVEDd of 35 mm. As this patient progressed toward PEA, LV systolic function was preserved with marked reduction of LV volumes with LVEDd of 10 mm at PEA, and filling being predominantly due to atrial contraction, with almost no blood entering during the rapid ventricular filling phase. However, after entering PEA, LV function slowly decreased over the next 15 min till asystole, and LV volumes remained minimal (Supplementary Video 1).

Patient 3 had apical akinesis with hypercontractile basilar portions of the left ventricle with LVEF of 45% and LVEDd of 37 mm. After cessation of life support, the basilar portions of the left ventricle gradually lost contractility, and LV volumes decreased. At PEA, LVEF was <10% with LVEDd of 25 mm. This persisted until asystole with minimal changes in LV systolic function and no significant change in LV volumes (Tables 2, 3; Supplementary Video 2).

**Table 2:** Patient's vitals and electrocardiographic findings during the observation period.

	**Blood pressure at disconnection of life support (mm of Hg)**	**Oxygen saturation at disconnection (%)**	**Lab work at life support withdrawal**	**Heart rate at the time of life support withdrawal (complexes/minute)**	**Heart rate at on the onset of PEA (complexes/minute)**
Patient 1	109/81	94	Normal	97	44
Patient 2	92/55	97	Normal	82	62
Patient 3	105/55	100	Normal	103	72

**Table 3:** Patient's electrocardiographic findings during the observation period.

	**EKG rhythm at life support withdrawal**	**EKG rhythm at PEA**	**Timeline to develop PEA (in minutes)**	**Systole present at PEA (on 2D-ECHO)**	**Time to develop asystole from PEA on 2D-ECHO (in minutes)**
Patient 1	NSR	Sinus bradycardia	10	YES	2
Patient 2	NSR	NSR	6	YES	15
Patient 3	Sinus tachycardia	NSR with wide QRS complex	9	YES	7

## Discussion

The modern approach to resuscitation was established in 1960 when the death rate from coronary artery disease was near its peak (5, 6). Then, VF was the predominant rhythm found, especially in pre-hospital groups (10, 11). PEA has become a more frequent presenting arrest rhythm to emergency medical services (EMS) and hospital providers (2, 3, 9), but treatment for PEA has undergone little change. As the demographics of cardiac arrest change, there must also be a shift in the focus of research in this field.

Animal and human studies have clearly elucidated the process through which a variety of hypoxic/anoxic insults lead to respiratory collapse, loss of consciousness, cardiovascular collapse, PEA, and ultimately asystole (9, 12–14). The EKG after collapse demonstrates bradycardic rhythms of a wide range of sources (PEA) for seconds to minutes until it decays to asystole. Arterial pressure waveforms illustrate the presence of contractions with levels of pressure too low for a pulse to be detected early in PEA (pseudo-PEA). Reversal of the stress prior to collapse results in rapid normalization of clinical status. After the collapse, in animal studies, support of breathing and/or circulation normalizes some but not all. With a longer delay to initiation of efforts, fewer animals can be resuscitated.

We have undertaken a long study of in-hospital resuscitation. Among the major findings has been the high rate of PEA and the realization that the process from respiratory or cardiovascular decompensation to PEA is a continuum, as in animal studies. Our large study of resuscitation in one hospital has provided a compelling look at the environment in which patients die and has allowed observation of hundreds of subjects entering PEA who either die or recover (9). We believe that the difficulty in studying PEA is similar to studying subatomic particles in physics; an ephemeral entity can only be studied in an environment where it can be identified as it emerges and followed to decay or resolution.

This small study, which combines EKG and echocardiographic assessments of patients who died without resuscitative efforts, supports the relevance of animal studies of death to humans. Each patient went from prolonged sinus bradycardia to various levels of AV block to asystole. EKG manifestations and contractions persisted many minutes after the pulse was lost. One patient had several isolated ventricular ectopic beats; however, none entered VF. The gradual weakening of pump function as demonstrated in the echocardiogram represents the different stages described as pseudo PEA, true PEA, and asystole. There were no sudden shifts during the progression, suggesting the researchers and clinicians who saw wall motion were looking at an earlier phase than those who saw no wall motion. In a resuscitation effort, the time allowed for echo observation is only a few seconds, and chest compressions and pressors affect the hemodynamic picture. In our subjects, wall motion decreased but never ceased until asystole. The distinction between pseudo-PEA and PEA is a function of brief looks at an evolving process; continuous observation of these few subjects demonstrates declining wall motion. The period of diastolic dysfunction was remarkably early, which led to the most impressive change; early and progressive decrease in diastolic volumes with increasing duration of PEA.

There are several limitations to the study. Only one model of PEA was studied with the presentation of only three patients. The ECHO studies were limited to one view, chosen to achieve continuous access rather than optimizing the ability to make measurements. Also, the effects of brain death on neural-cardiac feedback could also alter the nature and time-course of the cardiac response to systemic hypoxia and decline in blood pressure. The strengths of choosing this model are also relevant. Resuscitation efforts were not in progress to complicate the picture. Time was required to determine brain death and discuss transplant evaluation. The individuals had stable circulation with no pressors required. Studies of trauma, sepsis, and other models of PEA are needed.

The similarity of these findings to animal models suggests the development of therapy for animal studies may be relevant to human resuscitation. Animal models exist for the studies to use (14, 15). Increased diastolic dysfunction as time progresses during resuscitation is a major issue; future studies should explore the reversibility of this state. Cariporide, a selective inhibitor of the Na^+^/H^+^ exchanger isoform-1, decreased LV thickening in one animal study of VF arrest and enabled the generation of improved perfusion pressures with less depth of compression (16). Stimulation of pump function with post-extra-systolic pacing deserves exploration (17, 18) as does the potential for metabolic substrates to ameliorate the energy depletion associated with PEA. Reversal of PEA may be impossible in overwhelming insults, such as massive trauma, but the reversal of arrests from a drug overdose or volume overload could restore function to individuals who have good functional states.

Finally, we greatly appreciate the patients and family members, and the staff at The Medical Center for participating in this emotionally and ethically challenging study. We believe the value of the information will provide perspective on a poorly understood topic. In addition, our experience of no disruption of the perceived dying process by families supports the use of this model.

## Data Availability Statement

The original contributions presented in the study are included in the article/Supplementary Material, further inquiries can be directed to the corresponding author/s.

## Ethics Statement

The studies involving human participants were reviewed and approved by Institutional Review Boards of Mercer University School of Medicine and the Medical Center/Navicent Health. The patients/participants provided their written informed consent to participate in this study.

## Author Contributions

DP: conception and design. DP and HG: literature search and first draft. DP, HG, EJ, and FD: critical revision, editing, and final approval. All authors contributed to the article and approved the submitted version.

## Conflict of Interest

The authors declare that the research was conducted in the absence of any commercial or financial relationships that could be construed as a potential conflict of interest.

## Publisher's Note

All claims expressed in this article are solely those of the authors and do not necessarily represent those of their affiliated organizations, or those of the publisher, the editors and the reviewers. Any product that may be evaluated in this article, or claim that may be made by its manufacturer, is not guaranteed or endorsed by the publisher.

## References

[1] Ornato JP Peberdy MA. The mystery of bradyasystole during cardiac arrest. Ann Emerg Med. (1996) 27:576–87. 10.1016/S0196-0644(96)70160-9 8629778

[2] Herlitz J Andersson E Bång A Engdahl J Holmberg M lindqvist J. Experiences from treatment of out-of-hospital cardiac arrest during 17 years in Göteborg. Eur Heart J. (2000) 21:1251–8. 10.1053/euhj.2000.2150 10924315

[3] Keller SP Halperin HR. Cardiac arrest: the changing incidence of ventricular fibrillation. Curr Treat Options Cardiovasc Med. (2015) 17:392. 10.1007/s11936-015-0392-z 25981196 PMC4592695

[4] Myerburg RJ Halperin H Egan DA Boineau R Chugh SS Gillis AM. Pulseless electrical activity: definition, causes, mechanisms, management, and research priorities for the next decade: report from a national heart, lung, and blood institute workshop. Circulation. (2013) 128:2532–41. 10.1161/CIRCULATIONAHA.113.004490 24297818

[5] Dalen JE Alpert JS Goldberg RJ Weinstein RS. The epidemic of the 20(th) century: coronary heart disease. Am J Med. (2014) 127:807–12. 10.1016/j.amjmed.2014.04.015 24811552

[6] Parish DC Dinesh Chandra KM Dane FC. Success changes the problem: why ventricular fibrillation is declining, why pulseless electrical activity is emerging, and what to do about it. Resuscitation. (2003) 58:31–5. 10.1016/S0300-9572(03)00104-7 12867307

[7] Paradis NA Martin GB Goetting MG Rivers EP Feingold M Nowak RM. Aortic pressure during human cardiac arrest. Identification of pseudo-electromechanical dissociation. Chest. (1992) 101:123–8. 10.1378/chest.101.1.123 1729058

[8] Salen P Melniker L Chooljian C Rose JS Alteveer J Reed J. Does the presence or absence of sonographically identified cardiac activity predict resuscitation outcomes of cardiac arrest patients? Am J Emerg Med. (2005) 23:459–62. 10.1016/j.ajem.2004.11.007 16032611

[9] Parish DC Goyal H Dane FC. Mechanisms of death: there's more to it than sudden cardiac arrest. J Thorac Dis. (2018) 10:3081–7. 10.21037/jtd.2018.04.113 29997977 PMC6006107

[10] Bayes de Luna A Coumel P Leclercq JF. Ambulatory sudden cardiac death: mechanisms of production of fatal arrhythmia on the basis of data from 157 cases. Am Heart J. (1989) 117:151–9. 10.1016/0002-8703(89)90670-4 2911968

[11] Greene HL. Sudden arrhythmic cardiac death–mechanisms, resuscitation and classification: the Seattle perspective. Am J Cardiol. (1990) 65:4B−12B. 10.1016/0002-9149(90)91285-E 2404396

[12] Hanson JF Purks WK Anderson RG. Electrocardiographic studies of the dying human heart. Arch Intern Med. (1933) 51:965–77. 10.1001/archinte.1933.00150250149010

[13] Swann HG Brucer M. The sequence of circulatory, respiratory and cerebral failure during process of death–Its relation to resuscitability. Tex Rep Biol Med. (1951) 9:180–219. 14835460

[14] Redding JS Pearson JW. Resuscitation from asphyxia. JAMA. (1962) 182:283–6. 10.1001/jama.1962.03050420059015 13973494

[15] DeBehnke D. Resuscitation time limits in experimental pulseless electrical activity cardiac arrest using cardiopulmonary bypass. Resuscitation. (1994) 27:221–9. 10.1016/0300-9572(94)90036-1 8079056

[16] Kolarova JD Ayoub IM Gazmuri RJ. Cariporide enables hemodynamically more effective chest compression by leftward shift of its flow-depth relationship. Am J Physiol Heart Circ Physiol. (2005) 288:H2904–11. 10.1152/ajpheart.01181.2004 15708960

[17] Becker LC Levine JH DiPaula AF Guarnieri T Aversano T. Reversal of dysfunction in postischemic stunned myocardium by epinephrine and postextrasystolic potentiation. J.Am Coll. Cardiol. (1986) 7:580. 10.1016/S0735-1097(86)80468-5 3950238

[18] Heusch H Rose J Skyschally A Post H Schulz R. Calcium responsiveness in regional myocardial short-term hibernation and stunning in the *In Situ* porcine heart. Inotropic responses to postextrasystolic potentiation and intracoronary calcium. Circulation. (1996) 93:1556–66. 10.1161/01.CIR.93.8.1556 8608625

